# Low lordosis is a common finding in young lumbar disc herniation patients

**DOI:** 10.1186/s40634-020-00253-7

**Published:** 2020-05-31

**Authors:** Joel Beck, Helena Brisby, Adad Baranto, Olof Westin

**Affiliations:** Department of Orthopaedics, Institute of Clinical Sciences at Sahlgrenska Academy, Gothenburg University, and Sahlgrenska University Hospital, Bruna Straket 11b, S-413 45 Gothenburg, Sweden

**Keywords:** Spinal sagittal alignment, Lumbar disc herniation, Roussouly classification

## Abstract

**Purpose:**

The sagittal alignment of the lumbar spine and pelvis can be classified into several subtypes. It has been suggested that the risk of developing certain pathologies, such as a lumbar disc herniation (LDH) is affected by spinal sagittal profiles. The main aim of this study was to investigate the sagittal profile in young patients surgically treated for a lumbar disc herniation and if a discectomy would alter the sagittal parameters.

**Methods:**

Sixteen active young patients (mean age 18.3 ± 3.2 SD) with a lumbar disc herniation having a discectomy were included. A classification according to Roussouly of the sagittal parameters was made by two senior spinal surgeons, both pre-operatively and post-operatively on radiographs. The distribution of sagittal parameters and spinopelvic profiles were analysed and compared to a previous established healthy normal population.

**Results:**

This series of active young patients with LDH exhibited a low lumbar lordosis dominance, with Roussouly sagittal profiles type 1 and type 2 accounting for more than 75% of the examined patients. An analysis of the erect radiographs revealed no significant changes in the post-operative sagittal profile.

**Conclusions:**

This study showed that sagittal spinal alignment according to Roussouly in a young population with LDH is skewed compared with a normal population cohort. Furthermore, the lack of post-operative correction is suggestive of a non-ephemeral response to a LDH. Roussouly type 2 spinal sagittal profile may be a risk factor in young individuals suffering a disc herniation.

## Background

It has been hypothesised that certain spinal sagittal profiles might increase the risk of developing a lumbar disc herniation (LDH) [1]. A spinal profile with low lumbar lordosis “flat back” has been associated with an increased risk of disc degeneration at the L4-L5 and L5-S1 levels, and thus potentially predisposing these individuals to develop an early onset of disc degeneration [2, 3].

**Fig. 1:**
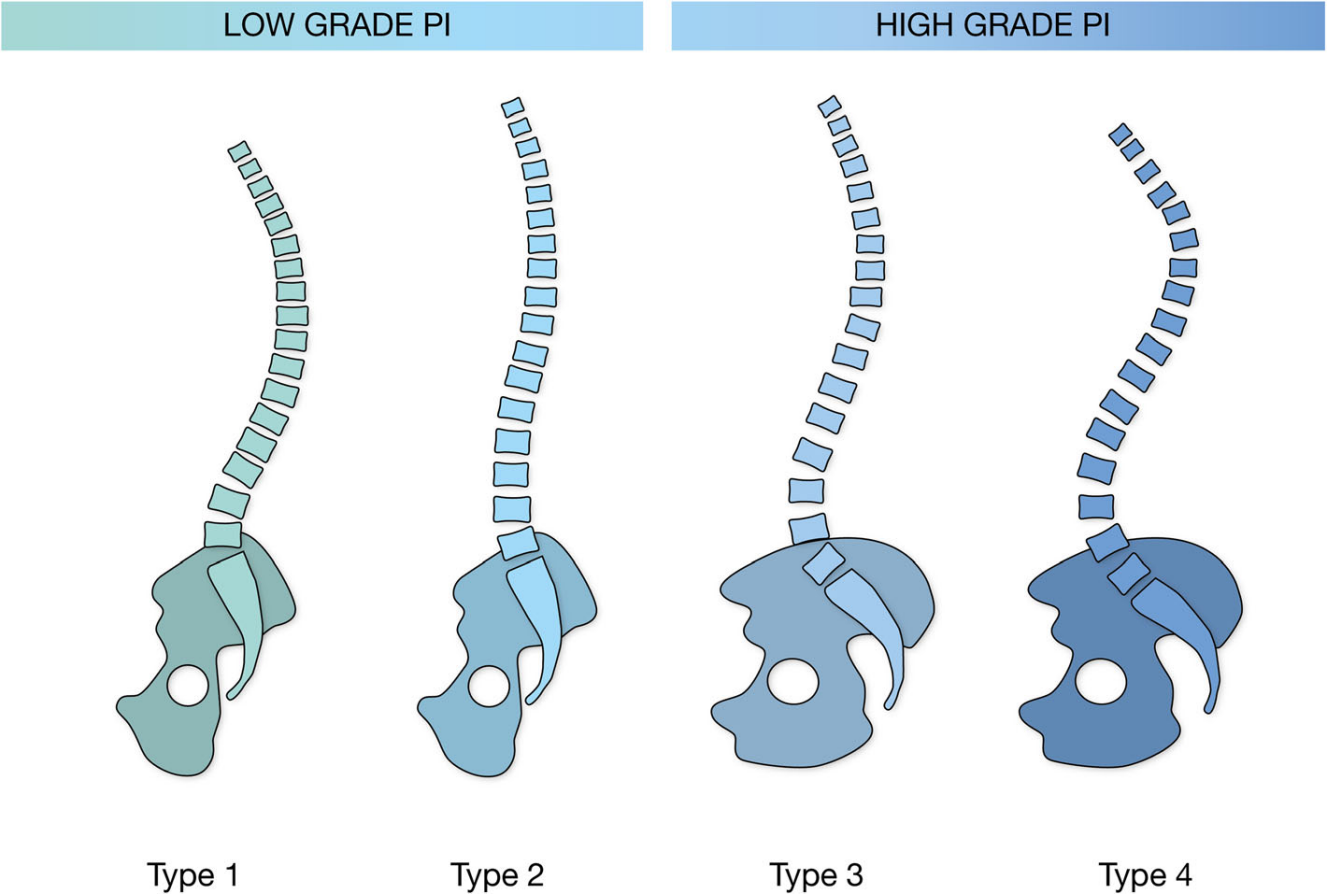
The four Roussouly subtypes of sagittal profile

The most commonly used classification for spinal morphology is the Roussouly classification, which divides the spinal subtypes into four different sagittal profiles, as illustrated in Fig. 1 [4].

Sagittal alignment, and the distribution of different sub-types in an unaffected population have been established in epidemiological studies by Mac-Thiong et al. [5]. It has been suggested in previous work that a lumbar disc herniation could affect the degree of lumbar lordosis [6]. Induced by unbearable pain, some patients are able to adjust their sagittal profile as a pain curtailing measure. In theory, recovery from the disc herniation-induced pain and back muscle spasm, either by surgical discectomy or conservative treatment, might therefore be able to adjust a temporary sagittal malalignment. Disc herniation surgery in an active young patient population is a procedure with very low incidence. In the general population, 20 LDH procedures per 100,000 inhabitants /year are performed in Sweden, Western Europe, whereas the incidence for young patients below the age of 20 is a low 0.6 per 100,000/year [7]. The scarcity of teenage patients presenting for surgery might reflect a conservative approach in general practise, and there is a potential for a substantial number of cases with delayed diagnosis. The aetiology and clinical course of LDH in children and adolescents differs from that in adults. Young individuals usually experience a longer overall pain duration, less sciatica and a higher prevalence of low back pain before the diagnosis of LDH is confirmed. One reason for this might be that the anatomical and morphological properties of lumbar discs in young patients differ significantly from those of mature discs in adults [8, 9].

The main aim of this study was to study the prevalence of an excessively low lumbar lordosis in young LDH patients requiring surgery. A secondary aim was to investigate the incidence of sagittal profile alterations following surgical intervention.

## Material and methods

### Patients

This is a series of young active patients with a diagnosed LDH that had to refrain from previous sport activities due to low back pain or sciatica. The patients were referred to a specialized sport/occupational spine unit at Sahlgrenska University Hospital, Gothenburg, Sweden 2014–2018 and prospectively enrolled in this small study series.

Inclusion criteria was a symptomatic lumbar disc herniation at the L4-L5 or L5-S1 level and failing < 12 weeks physiotherapeutic or other non-operative treatment. Age exclusion was arbitrarily set to < 25 years, to minimise the impact of age-related lumbar disc and facet joint degeneration affecting sagittal spinal alignment.

### Imaging examinations

A pre-operative and post-operative erect radiograph with an anteroposterior (AP) and lateral view were obtained and reviewed to evaluate sagittal spinopelvic parameters. This erect radiograph of the spine visualised the entire spine from the upper cervical spine to the proximal part of the femurs, including the hip joints. This radiological protocol was performed in accordance with previous studies examining spinopelvic parameters [10–12]. Classification of the sagittal alignment and measurements of thoracic kyphosis (TK), lumbar lordosis (LL), sacral slope (SS), pelvic tilt (PT) and pelvic incidence (PI) and corresponding to Roussouly et al. in the four classical subtypes were independently performed by two senior spinal surgeons using blinded radiographs [13]. Radiographic assessments were performed by exporting the patient’s radiological material to Surgimap (Surgimap, 475 Park Ave S, 11th Fl, New York, NY 10016, USA). All patients were examined with at least one 1.5 T or 3.0 T MRI investigation pre-operatively in a machine to confirm the diagnosis.

### Surgical procedure

All the patients included in the present study underwent surgical lumbar discectomy. This procedure is frequently performed in modern clinical practice and the minimal invasive surgical methods have been published in several articles [14, 15].

### Statistics and data analysis

The data from the radiographic measurements were exported to IBM Statistical Package for Social Science SPSS (IBM SPSS Statistics for Mac, Version 24.0. Armonk, NY IBM Corp.) for evaluation. The data were statistically described in terms of mean and standard deviation (SD), median and range, or frequencies and percentages, when appropriate. Student’s t-test was used to determine the significant differences between pre- and post-operative X-rays. Pearson’s correlation coefficient was used to compare the correlation between the sagittal parameters in the pre- and post-operative erect radiographs. A two-sample proportion comparison was carried out using the two-sample z-test. All tests were two-sided and significance was set at *p* < 0.05 for each test. The changes in radiographic parameters were expressed as (degrees±SD). The inter-rater reliability of the measurements was determined with the intraclass correlation coefficient (ICC, 2,1) (two-way random model, absolute agreement, single measurements). To categorize the level of agreement among ICC values, we used the classification system proposed by Fleiss (1979). ICC values of less than 0.40 represent poor, values between 0.4 and 0.75 represent fair to good, while values above 0.75 represent excellent reliability.

## Results

### Patient metrics and baseline characteristics

Background and demographic data for the patient population are presented in Table 1. More women (*n* = 12) than men (*n* = 4) were recruited to the present series (63% vs 37%). Four of the patients had LDH at the L4-L5 level, whereas 12 had LDH at the L5-S1 level.

**Table 1:** Study population demographic data. Percentage and (absolute numbers)

Gender	men 37% (4)
	women 63% (12)
Age	mean 18.3 (range 14–23)
Level of surgery	L4-L5 37.5% (6)
	L5-S1 62.5% (10)
Duration of sciatica	18 months (range 3–36)

### Interobserver correlation of radiographic measurements

An analysis of the radiographic assessment revealed a high level of agreement in inter-observer measurements, with low variability. Intraclass correlation coefficients for interobserver pelvic measures were between 0.75 and 0.95, indicating excellent agreement as seen in Table 2.

**Table 2:** Inter-observer analysis of radiographic measurements. Intraclass correlation coefficient with good (> 0.75) or excellent (> 0.90) reproducibility. CI = Confidence Interval

Measurements	Intraclass correlation	95% CI	*p*-value
Pre-operative measurements			
Sacral slope	0.86	0.64–0.95	< 0.001
Pelvic incidence	0.75	0.64–0.91	< 0.001
Pelvic tilt	0.89	0.73–0.96	< 0.001
Lumbar lordosis	0.94	0.79–0.97	< 0.001
Pelvic incidence – Lumbar lordosis	0.93	0.83–0.98	< 0.001
Post-operative measurements			
Sacral slope	0.76	0.64–0.95	< 0.001
Pelvic incidence	0.82	0.54–0.94	< 0.001
Pelvic tilt	0.93	0.79–0.97	< 0.001
Lumbar lordosis	0.9	0.72–0.96	< 0.001
Pelvic incidence – Lumbar lordosis	0.95	0.87–0.98	< 0.001

### Sagittal spinal measurements

The PI was constant when comparing pre and postoperative measurements for all individual patients and all potential subsequent alterations in LL were thus related to alterations in PT and SS. However, None of the alterations in the spinal sagittal parameters following surgery were statistically significant and furthermore, all sagittal profile changes in Table 3 were within ±5 degrees.

**Table 3:** Sagittal measurements. Patients grouped according Roussouly et Al, pre- and post-operative median measurements with (range)

Roussouly	1		2		3		4	
	Pre	Post	Pre	Post	Pre	Post	Pre	Post
Pelvic tilt	11.5 (28)	11.5 (23)	15 (24)	13.5 (20)	10 (16)	6 (2)	0 (3)	3 (6)
Sacral Slope	28.0 (17)	30.5 (12)	29.5 (10)	31 (11)	36.5 (3)	41 (8)	47 (4)	44.5 (1)
Lumbar lordosis	43.5 (25)	48.0 (16)	45 (14)	47 (7)	56 (4)	56.5 (1)	67.5 (3)	67 (10)
Pelvic incidence	45 (20)		46 (20)		47 (10)		47.5 (7)	

### Distribution of sagittal profiles

**Fig. 2:**
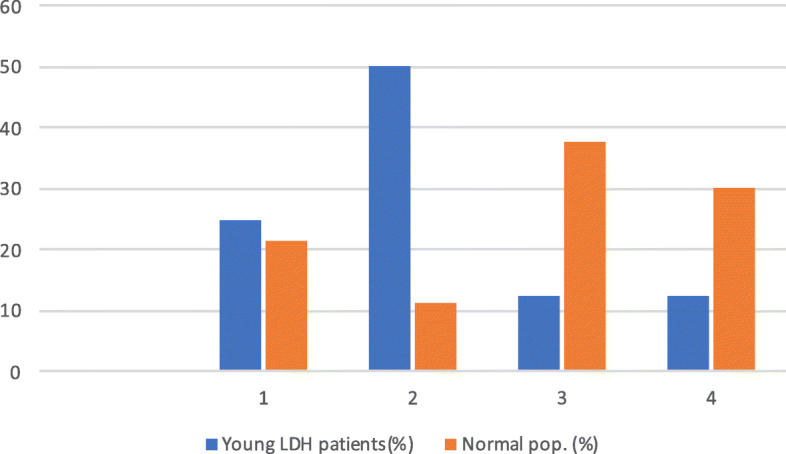
Distribution of the patients across the four Roussouly subtypes

 Within the cohort, the distribution of spinal profiles was skewed when compared to a normal population. An increased proportion of type 1, and especially type 2 spinal sagittal profile could be observed, as stated in Fig. 2.

## Discussion

The principal finding in the present study was that a majority (75%) of young active LDH patients having surgery had a low PI and significantly lower LL than a comparable normal population when compared to Mac-Thiong et Al [16]. Furthermore, the proportions of sagittal profiles in the current study population was skewed towards type 1 (25%) or type 2 (50%) (global thoracolumbar kyphosis with a short hyper-lordotic segment and “flat back” respectively), and showing an inverted distribution when compared to a normal unaffected comparable population [17]. In summary, the young patients with LDH were more than twice as likely to have a very low LL than the normal population.

All surgeries were performed at the two lowermost levels of the lumbar spine, which is consistent with the situation in large epidemiological reports where these two lumbar levels account for more than 97% of LDH surgeries [18].

The sagittal profile in the studied patients appeared not to be induced by transient pain or muscle spasm since no major changes of the sagittal parameters were seen when pre and postoperative measurements were compared. On the contrary, the observed sagittal profiles could be interpreted as a predisposing factor in the development of LDH in this population. This is in line with previous reports where low LL spinal morphology have been demonstrated to predispose for an early disc degeneration [1, 2, 19–21]. The type 1 or type 2 spinal sagittal alignment may increase the risk for these individuals to develop LDH by a lack of lordosis in the lower part of the lumbar spine [22]. Based on the results in our cohort of young active LDH patients, it can be hypothesised that the “flat” LL shifts the vertical load distribution towards the disc complex, potentially affecting the mechanical integrity of the disc. Further support for this theory can be found in previous studies which examine the incidence of lumbar disc degeneration and LDH in physically active young adults and adolescents performing sports with excessive vertical loads [23, 24].

The studied patient cohort had a mean age of 18.3 (range 14–24) at the time of surgery. Our cohort contained a significant number of very active patients that had endured a prolonged course of non-successful conservative treatment. Despite not being able to exercise due to back pain pre-operatively, the patients stated that they could resume a previous activity level following surgery, and 6 of the patients could return to sub-professional sports. This is an indication that active young patients improve despite having endured a long period of pre-operative symptoms, and are able to regain or even improve their previous fitness level. The development of lumbar lordosis in the human spine is an age-dependent transformation and further research should be directed towards assessing the development of LL and skeletal maturity in young adolescents with spine and back pain problems, since a “flat back” sagittal profile in adolescence in our study could be a significant risk factor for developing a LDH.

To our knowledge this is the first study to evaluate the sagittal profile in a series of young active patients with lumbar disc herniations requiring surgery. Secondly, we also measured the sagittal profile post-surgery to evaluate alterations following surgery and recovery.

The limitations of the current study are the very limited number of young LDH patients presenting for surgery, but, since this is a rare condition in this age group, large sample sizes are difficult to obtain. A second limitation is that the study only evaluates patients with ongoing back- or radicular pain (sciatica), requiring surgery and hence doesn’t include patients managed with conservative treatment.

## Conclusion

In conclusion, this study showed that the sagittal spinal alignment in a young active patient population with LDH needing surgery is skewed compared with a normal population cohort and it is not affected/altered by the disc herniation disease in itself. Our findings support the theory that young individuals without previous spinal degenerative changes and having an excessively low LL are exposed to an increased load on the anterior part of the spinal column and subsequently have a higher risk of suffering premature lumbar disc degeneration and LDH.

## Data Availability

The datasets used and analyzed during the current study are available from the corresponding author on reasonable request.

## Abbreviations

LDH: Lumbar Disc Herniation
PI: Pelvic Incidence
PT: Pelvic Tilt
SS: Sacral Slope
TK: Thoracic kyphosis
LL: Lumbar Lordosis
AP: Anteroposterior
SD: Standard Deviation
ICC: Intraclass Correlation Coefficient
